# Predicting efficacy of combined functional electrical stimulation combined with mirror therapy in post-stroke lower limb dysfunction: development and validation of a nomogram model

**DOI:** 10.3389/fneur.2025.1653797

**Published:** 2025-12-05

**Authors:** Wei Wang, Guobin Zhao, Genchun Guo

**Affiliations:** 1 Sport and Health College of Shinhan University, Uijeongbu-si, Gyeonggi-do, Republic of Korea; 2 Department of Rehabilitation Medicine, Affiliated Hospital 6 of Nantong University (Yancheng Third People’s Hospital), Yancheng, China

**Keywords:** cerebral infarction, lower limb motor dysfunction, functional electrical stimulation mirror therapy, logistic analysis, nomogram prediction model

## Abstract

**Objective:**

To investigate the predictive factors of the efficacy of conventional therapy combined with functional electrical stimulation combined with mirror therapy (FES-MT) in the treatment of lower limb dysfunction after cerebral infarction, and to construct and validate a nomogram prediction model to assist in clinical precision treatment.

**Methods:**

A total of 212 patients with lower limb dysfunction after cerebral infarction, who received the aforementioned treatment, were enrolled retrospectively. Based on therapeutic efficacy, they were divided into an effective group and an ineffective group. Relevant clinical indicators were collected. Patients were randomly assigned in a 7:3 ratio to a training set (*n* = 148) and a validation set (*n* = 64). In the training set, multivariate logistic regression analysis was performed to identify independent influencing factors, and a nomogram prediction model was constructed. The predictive performance of the model was evaluated using calibration curves, the concordance index (C-index), and decision curve analysis.

**Results:**

Among the 212 patients, 142 were classified as effective and 70 as ineffective. Multivariate logistic regression analysis showed that longer time from onset to treatment, higher fasting plasma glucose, higher homocysteine, larger infarct volume, higher National Institutes of Health Stroke Scale score, and lower pre-treatment Fugl-Meyer score (lower limb) were independent risk factors for ineffective treatment. The constructed nomogram model exhibited C-index values of 0.890 and 0.872 in the training and validation sets, respectively, with mean absolute errors of 0.124 and 0.114. In the Hosmer-Lemeshow test, the χ^2^ values were 9.907 (*p* = 0.271) for the training set and 7.194 (*p* = 0.515) for the validation set. Receiver operating characteristic curve analysis showed that the area under the curve values were 0.890 (95% CI: 0.822–0.957) and 0.872 (95% CI: 0.747–0.998) for the training and validation sets, respectively, with combined sensitivity and specificity of 0.847, 0.875 and 0.812, 0.750.

**Conclusion:**

The nomogram prediction model, based on the identified influencing factors, demonstrates excellent predictive performance for the clinical efficacy of conventional therapy combined with FES-MT in patients with lower limb dysfunction after cerebral infarction. This model facilitates clinical outcome prediction and aids in formulating individualized treatment strategies.

## Introduction

Ischemic stroke was a prevalent cerebrovascular disease characterized by high incidence, disability, and mortality rates (1). Its pathogenesis primarily involved the interruption of cerebral blood flow, most commonly due to thrombosis or embolism, leading to ischemic brain injury and neuronal death. This process results in a spectrum of neurological deficits, among which lower limb motor dysfunction was one of the most common sequelae. This dysfunction significantly impaired patients’ ability to perform daily activities and diminishes quality of life (2, 3). The management of stroke and its long-term consequences posed a substantial challenge to modern healthcare systems, consuming considerable resources and emphasizing the critical need for effective rehabilitation strategies to improve functional outcomes and reduce societal burden. Current conventional therapies, such as pharmacological interventions and rehabilitation training, serve as fundamental approaches to symptom amelioration, yet their efficacy varies considerably among individuals (4). Functional electrical stimulation combined with mirror therapy (FES-MT), an emerging rehabilitation strategy, promotes the recovery of lower limb motor function to some extent by eliciting muscle contractions via electrical stimulation and augmenting neural reorganization through mirror-induced visual feedback (5). However, clinical outcomes of this combined regimen exhibit interpatient heterogeneity due to the influence of multiple factors.

To date, research on the predictive factors influencing the clinical efficacy of conventional therapy combined with FES-MT in ischemic stroke patients with lower limb dysfunction remains insufficient, and a precise predictive model was lacking. The nomogram model, capable of integrating multifactorial predictors to visually estimate event probabilities, has been widely employed in medical research. This study aims to develop and validate a nomogram prediction model based on relevant influencing factors, thereby providing clinicians with a novel tool to evaluate therapeutic outcomes, tailor individualized treatment plans, and enhance therapeutic precision and efficacy, ultimately improving patient prognosis.

## Materials and methods

### Study population

This retrospective study analyzed data from 212 patients with lower limb dysfunction after cerebral infarction, who were hospitalized and received the combined therapy (conventional therapy + FES-MT) between July 2023 and January 2025. The patients were randomly divided into a training set (*n* = 148) and a validation set (*n* = 64) at a ratio of 7:3. The study was approved by the Ethics Committee of Yancheng Third People’s Hospital (No. 2021-May-2746), and informed consent was obtained from all patients. Clinical trial number: not applicable.

### Patient inclusion and adherence

The inclusion criteria were as follows: (1) presence of lower limb dysfunction (operationally defined using the Fugl-Meyer Assessment for Lower Extremity (FMA-LE) (6), a validated post-stroke motor function scale with a full score of 66; dysfunction was confirmed if the FMA-LE score was<66) within 6 months post-onset; (2) completion of the entire 8-week the combined therapy (conventional therapy + FES-MT); (3) age 18–80 years; (4) signed informed consent obtained from either the patient or legal guardian. The exclusion criteria included: (1) severe dysfunction of vital organs (e.g., heart, liver, or kidneys); (2) diagnosis of malignant tumors; (3) psychiatric disorders (e.g., schizophrenia, major depressive disorder diagnosed by a psychiatrist) or cognitive impairment [defined as Mini-Mental State Examination (MMSE) score < 24 or Montreal Cognitive Assessment (MoCA) score < 26] preventing compliance with treatment/assessment; (4) receipt of any specialized therapy affecting lower limb motor recovery (including intensive physiotherapy (≥5 sessions/week, >60 min/session), robotic rehabilitation, transcranial magnetic stimulation (TMS), repetitive TMS) within the preceding 3 months; (5) patients who were lost to follow-up, did not complete the full treatment course, or had significant missing data. A total of 250 patients were initially screened during the study period. Of these, 38 patients were excluded: 15 were lost to follow-up, 12 did not complete the 8-week protocol due to personal reasons or transfer to another hospital, and 11 had incomplete medical records. Consequently, 212 patients (84.8% of the initially screened cohort) who demonstrated full adherence to the protocol and had complete datasets were included in the final analysis.

### Therapeutic interventions

All patients received guideline-based pharmacological management, including: Anti-platelet aggregation therapy: Aspenter (Enteric-coated Aspirin Tablets, 100 mg once daily) or Clopidogrel (75 mg once daily). Lipid-modifying therapy: Atorvastatin (20 mg once nightly) or Rosuvastatin (10 mg once nightly). Management of underlying conditions: Antihypertensive agents (e.g., Amlodipine, Valsartan), hypoglycemic agents (e.g., Metformin, Insulin), or other medications were prescribed as needed to maintain blood pressure and blood glucose within target ranges.

Concurrently, a standardized rehabilitation program was administered to all patients by licensed physical therapists, 5 days per week for 8 weeks. Each daily session lasted approximately 60 min and included: Range of motion exercises: Passive and active-assistive exercises for the paralyzed lower limb (10 min). Muscle strength training: Resistance training for key muscle groups of the lower limbs (e.g., quadriceps, hamstrings, glutei) using elastic bands or weight cuffs (20 min).

Balance training: Static and dynamic balance exercises in sitting and standing positions (15 min). Gait training: Over-ground walking practice with appropriate assistive devices (e.g., ankle-foot orthosis, walker) and therapist assistance as needed (15 min).

In addition, FES-MT was implemented. A functional electrical stimulator was used with electrodes attached to the major muscle groups of the affected lower limb (quadriceps femoris, hamstrings, tibialis anterior). The current intensity was adjusted based on patient tolerance (stimulation frequency: 30 Hz; duration: 20 min/session; once daily). During stimulation, patients faced a mirror and performed identical movements with the unaffected limb, utilizing visual feedback from the mirror to enhance motor awareness of the affected limb. The FES-MT protocol was conducted 5 days/week for 8 weeks, which was referenced and adapted from a previous randomized controlled trial on post-stroke rehabilitation (5).

### Data collection and assessment methods

Demographic and clinical characteristics were collected, including age, sex, body mass index (BMI), smoking history (yes/no), alcohol consumption (yes/no), medical history of hypertension (yes/no), diabetes mellitus (yes/no), hyperlipidemia (yes/no), and time from onset to treatment. Fasting plasma glucose (FPG), glycated hemoglobin (HbA1c), lipid profiles including total cholesterol (TC), triglycerides (TG), low-density lipoprotein cholesterol (LDL-C), high-density lipoprotein cholesterol (HDL-C), homocysteine (Hcy), and high-sensitivity C-reactive protein (hs-CRP) were measured using an automated biochemical analyzer.

Imaging parameters: Infarct volume and lesion location were assessed via cranial magnetic resonance imaging (MRI). All examinations were performed using a 3.0 T MRI scanner (Siemens Magnetom Prisma) with a standard head coil. T2-weighted fluid-attenuated inversion recovery (T2-FLAIR) sequences were utilized-due to their high sensitivity in delineating ischemic lesions in the subacute and chronic phases-for both infarct volume quantification and lesion localization. Two experienced radiologists, who were blinded to the clinical outcomes and group assignments of the patients, independently performed two tasks: (1) Manually outlining the boundary of the infarct area on each slice of the T2-FLAIR images using the ITK-SNAP software (version 3.8.0); the software automatically calculated the total infarct volume (in cm^3^) based on the outlined regions and slice thickness, with the final volume taken as the average of the two radiologists’ measurements to enhance accuracy. (2) Recording lesion location according to brain regions closely related to lower limb motor function, including the basal ganglia, internal capsule, primary motor cortex, and brainstem. Disagreements between radiologists were resolved through joint consultation to ensure consistency.

Neurological and motor function assessments: Neurological deficits were evaluated by licensed neurologists (with ≥3 years of stroke clinical experience) using the National Institutes of Health Stroke Scale (NIHSS) (7). Lower limb motor function, balance, and functional mobility were assessed pre- and post-treatment by licensed physical therapists who had received standardized training in the FMA-LE, Berg Balance Scale (BBS), and Functional Independence Measure (FIM). All assessors were blinded to the patients’ treatment grouping (training/validation set) and outcome expectations (effective/ineffective group) to minimize observer bias.

### Therapeutic efficacy evaluation

After 8 weeks of treatment, to fully reflect patients’ comprehensive functional recovery (including key dimensions such as motor function, balance, and daily autonomy), therapeutic outcomes were classified based on multi-domain assessments-with changes in FMA-LE scores as the core indicator, supplemented by balance function assessment (using the Berg Balance Scale, BBS, a standard tool for post-stroke balance evaluation in clinical practice) and functional mobility assessment (focusing on gait-related autonomy, e.g., independent walking or stair-climbing ability, which is closely related to daily living activities): It is important to note that the minimal clinically important difference (MCID) of FMA-LE-an indicator to distinguish “clinically meaningful recovery” from “insignificant changes”-is 4–5 points, which conforms to widely accepted clinical rehabilitation guidelines in the stroke rehabilitation field.

For FMA-LE-based efficacy classification: (1) Marked improvement: FMA-LE score increase≥50% from baseline. (2) Effective: FMA-LE score increase of 20–49% from baseline. (3) Ineffective: FMA-LE score improvement<20%, no change, or deterioration.

Patients with marked improvement or effective outcomes were combined into an “effective group,” while the ineffective cases comprised the “ineffective group.”

### Statistical analysis

All statistical analyses were performed using R 4.0.3 software. The following R packages were employed: Table 1 for creating (baseline characteristics), rms for conducting logistic regression analysis and constructing the nomogram, pROC for performing receiver operating characteristic (ROC) curve analysis and calculating the area under the curve (AUC), Resource Selection for the Hosmer-Lemeshow goodness-of-fit test, and rmda for decision curve analysis (DCA). Quantitative data with a normal distribution were expressed as mean±standard deviation, and intergroup comparisons were conducted using independent samples t-tests. Categorical data were presented as numbers and percentages (%), and comparisons between groups were performed usingχ^2^ test or Fisher’s exact test, as appropriate. Univariate analysis was first performed to screen for potential influencing factors associated with treatment outcome (ineffective vs. effective). Variables that demonstrated statistical significance (*p* < 0.05) in the univariate analysis were subsequently incorporated into a multivariate logistic regression analysis using the rms package to identify independent predictors. The results were presented as odds ratios (ORs) with corresponding 95% confidence intervals (CIs). Variance inflation factors (VIF) were calculated to exclude multicollinearity (VIF threshold <10). A nomogram prediction model was constructed based on the results of the multivariate logistic regression analysis to visually estimate the probability of ineffective treatment for individual patients. The predictive performance of the nomogram was evaluated in both the training and validation sets. Discrimination was assessed using the concordance index (C-index), which is equivalent to the AUC, and visually represented by ROC curves. Calibration, which refers to the agreement between predicted probabilities and observed outcomes, was assessed using calibration curves and the Hosmer-Lemeshow test. Clinical utility was evaluated using DCA. A two-tailed *p*-value<0.05 was considered statistically significant.

**Table 1:** Comparison of baseline characteristics between training and validation sets.

Indicators		Training set (*n* = 148)	Validation set (*n* = 64)	χ^2^/*t*	*P*
Age (years)		62.54 ± 9.36	61.87 ± 10.52	0.460	0.645
Sex	Male	81 (54.73)	36 (56.25)	0.041	0.838
	Female	67 (45.27)	28 (43.75)		
BMI (kg/m^2^)		24.68 ± 3.23	24.47 ± 3.42	0.426	0.669
Smoking history	Yes	46 (31.08)	18 (28.13)	0.185	0.666
	No	102 (68.92)	46 (71.88)		
Alcohol consumption	Yes	39 (26.35)	13 (20.31)	0.880	0.348
	No	109 (73.65)	51 (79.69)		
Hypertension	Yes	72 (48.65)	29 (45.31)	0.199	0.655
	No	76 (51.35)	35 (54.69)		
Diabetes mellitus	Yes	41 (27.70)	16 (25.00)	0.166	0.683
	No	107 (72.30)	48 (75.00)		
Hyperlipidemia	Yes	52 (35.14)	22 (34.38)	0.011	0.915
	No	96 (64.86)	42 (65.62)		
Onset-to-treatment time (days)		10.58 ± 3.56	9.87 ± 3.84	1.301	0.149
FPG (mmol/L)		5.87 ± 1.23	5.79 ± 1.31	0.426	0.670
HbA1c (%)		6.34 ± 0.87	6.19 ± 0.92	1.132	0.258
TC (mmol/L)		4.92 ± 0.95	4.73 ± 1.07	1.286	0.199
TG (mmol/L)		1.78 ± 0.64	1.72 ± 0.71	0.606	0.545
LDL-C (mmol/L)		3.01 ± 0.78	2.84 ± 0.86	1.411	0.159
HDL-C (mmol/L)		1.28 ± 0.24	1.26 ± 0.28	0.529	0.597
Hcy (μmol/L)		15.67 ± 4.56	15.23 ± 4.82	0.633	0.526
hs-CRP (mg/L)		3.56 ± 1.23	3.48 ± 1.31	0.426	0.670
Infarct volume (cm^3^)		15.23 ± 6.54	14.89 ± 6.82	0.343	0.731
Lesion location	Basal ganglia	59 (39.86)	26 (40.63)	0.026	0.999
	Internal capsule	37 (25.00)	16 (25.00)		
	Primary motor cortex	30 (20.27)	13 (20.31)		
	Brainstem	22 (14.86)	9 (14.06)		
NIHSS		15.00 ± 4.00	14.00 ± 3.00	1.792	0.074
Pre-treatment Fugl-Meyer Assessment (lower extremity)		26.00 ± 8.00	24.00 ± 9.00	1.608	0.109

## Results

### Baseline characteristics of patients in training and validation sets

Among 212 enrolled patients, therapeutic efficacy was effective in 142 cases (66.98%) versus 70 cases (33.02%) with ineffective. The training set comprised 148 patients (104 effective, 44 ineffective), while the validation set included 64 patients (38 effective, 26 ineffective). No statistically significant differences were observed between the two Sets regarding baseline characteristics, including age, gender, BMI, smoking history, alcohol consumption, comorbidities, hematological parameters, neuroimaging indices, or neurological function assessments (all *p* > 0.05) (Table 1).

### Analysis of influencing factors for treatment efficacy in the training set

Univariate analysis of the training set revealed that there were statistically significant differences (*p* < 0.05) between patients with effective treatment and those with ineffective treatment in terms of age, time from onset to treatment, FPG, Hcy, infarct volume, NIHSS score, and pre-treatment Fugl-Meyer assessment (lower limb) (Table 2). With treatment efficacy (effective = 0, ineffective = 1) as the dependent variable and factors with *p* < 0.05 in the univariate analysis as covariates, a multivariate Logistic regression analysis was conducted. The results indicated that time from onset to treatment, FPG, Hcy, infarct volume, NIHSS score, and pre-treatment Fugl-Meyer score (lower limb) were independent influencing factors for treatment outcome (all *p* < 0.05) (Table 3). Longer time from onset to treatment, higher FPG, higher Hcy, larger infarct volume, and higher NIHSS score were risk factors for ineffective treatment. A higher pre-treatment Fugl-Meyer assessment (lower limb) was a protective factor. In the regression model, the tolerance of each variable was > 0.1, the VIF was < 10, and the condition index was < 30. Moreover, there was no situation where the variance proportion of multiple covariates under the same eigenvalue was > 50%. Therefore, there was no collinearity among the covariates. Through statistical testing, no significant interaction effects among the factors were found, suggesting that in this study, the effects of each factor on treatment efficacy were relatively independent (Table 4).

**Table 2:** Univariate analysis of influencing factors for treatment efficacy in the training set.

Indicators		Ineffective group (*n* = 44)	Effective group (*n* = 104)	χ^2^/t	*P*
Age (years)		65.23 ± 9.34	60.17 ± 9.21	3.042	0.002
Sex	Male	25 (56.82)	56 (53.85)	0.110	0.739
	Female	19 (43.18)	48 (46.15)		
BMI (kg/m^2^)		24.78 ± 3.31	24.63 ± 3.17	0.259	0.795
Smoking history	Yes	13 (29.55)	33 (31.73)	0.068	0.792
	No	31 (70.45)	71 (68.27)		
Alcohol consumption	Yes	11 (25.00)	28 (26.92)	0.058	0.808
	No	33 (75.00)	76 (73.08)		
Hypertension	Yes	19 (43.18)	53 (50.96)	0.749	0.386
	No	25 (56.82)	51 (49.04)		
Diabetes mellitus	Yes	12 (27.27)	29 (27.88)	0.005	0.939
	No	32 (72.73)	75 (72.12)		
Hyperlipidemia	Yes	16 (36.36)	36 (34.62)	0.041	0.838
	No	28 (63.64)	68 (65.38)		
Onset-to-treatment time (days)		12.46 ± 3.62	9.83 ± 3.41	4.210	0.001
FPG (mmol/L)		6.32 ± 1.31	5.57 ± 1.19	3.400	0.001
HbA1c (%)		6.49 ± 0.87	6.28 ± 0.76	1.470	0.143
TC (mmol/L)		4.98 ± 0.98	4.89 ± 0.93	0.529	0.597
TG (mmol/L)		1.81 ± 0.66	1.74 ± 0.62	0.615	0.539
LDL-C(mmol/L)		3.08 ± 0.82	2.97 ± 0.76	0.786	0.433
HDL-C(mmol/L)		1.27 ± 0.26	1.28 ± 0.23	0.232	0.816
Hcy (μmol/L)		17.32 ± 4.78	14.89 ± 4.37	3.006	0.003
hs-CRP (mg/L)		3.62 ± 1.28	3.52 ± 1.19	0.456	0.648
Infarct volume (cm^3^)		17.94 ± 6.89	13.31 ± 6.32	3.965	0.001
Lesion location	Basal ganglia	18 (40.91)	41 (39.42)	1.739	0.628
	Internal capsule	10 (22.73)	27 (25.96)		
	Primary motor cortex	9 (20.45)	21 (20.19)		
	Brainstem	7 (15.91)	15 (14.42)		
NIHSS		17.00 ± 4.00	15.00 ± 3.00	3.343	0.001
Pre-treatment Fugl-Meyer Assessment (lower extremity)		22.00 ± 8	28.00 ± 7.00	4.564	0.001

**Table 3:** Variable assignment methods.

Variable	Meaning	Assignment
X1	Age	Continuous variable
X2	Time from onset to treatment	Continuous variable
X3	FPG	Continuous variable
X4	Hcy	Continuous variable
X5	Infarct volume	Continuous variable
X6	NIHSS	Continuous variable
X7	Pre-treatment Fugl-Meyer Assessment (lower extremity)	Continuous variable
Y	Treatment efficacy	Effective = 0, Ineffective = 1

**Table 4:** Multivariate Logistic regression analysis of influencing factors for treatment effects in the training set.

Indicators	B	*SE*	Wald	*P*	OR	95%CI
Age	0.042	0.026	2.627	0.105	1.043	0.991–1.096
Time from onset to treatment	0.240	0.078	9.493	**0.002**	**1.271**	**1.091–1.480**
FPG	0.411	0.200	4.232	**0.040**	**1.509**	**1.020–2.232**
Hcy	0.125	0.055	5.130	**0.024**	**1.133**	**1.017–1.262**
Infarct volume	0.105	0.040	6.804	**0.009**	**1.110**	**1.026–1.201**
NIHSS	0.279	0.082	11.498	**0.001**	**1.321**	**1.125–1.552**
Pre-treatment Fugl-Meyer Assessment (lower extremity)	−0.070	0.033	4.500	**0.034**	**0.933**	**0.875–0.955**

Bold values indicate that the statistical test results of the corresponding variables are statistically significant (*p* < 0.05), which means these variables are independent influencing factors for the ineffectiveness of the combined therapy (FES-MT) in treating post-stroke lower limb dysfunction.

### Construction of the nomogram prediction model

**Figure 1:**
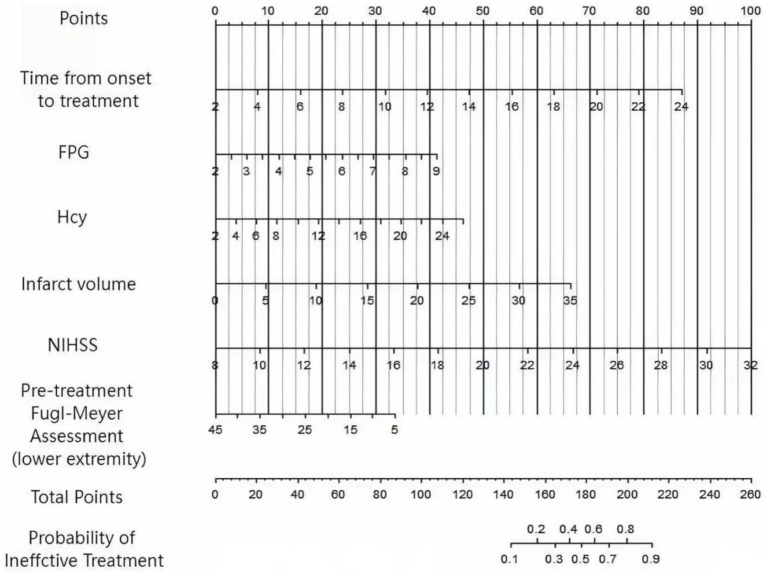
Nomogram prediction model diagram for the treatment effect of lower limb dysfunction in cerebral infarction constructed based on multiple factors.

Based on the independent influencing factors identified by multivariate Logistic regression analysis, a Nomogram prediction model was constructed for the clinical efficacy of the treatment of patients with lower limb dysfunction after cerebral infarction using the combination of conventional treatment regimens and FES-MT training. According to the regression coefficients of each factor, corresponding score scales were assigned to each factor. The total score corresponded to the probability of ineffective treatment for patients (Figure 1).

### Evaluation and validation of the nomogram prediction model

**Figure 2:**
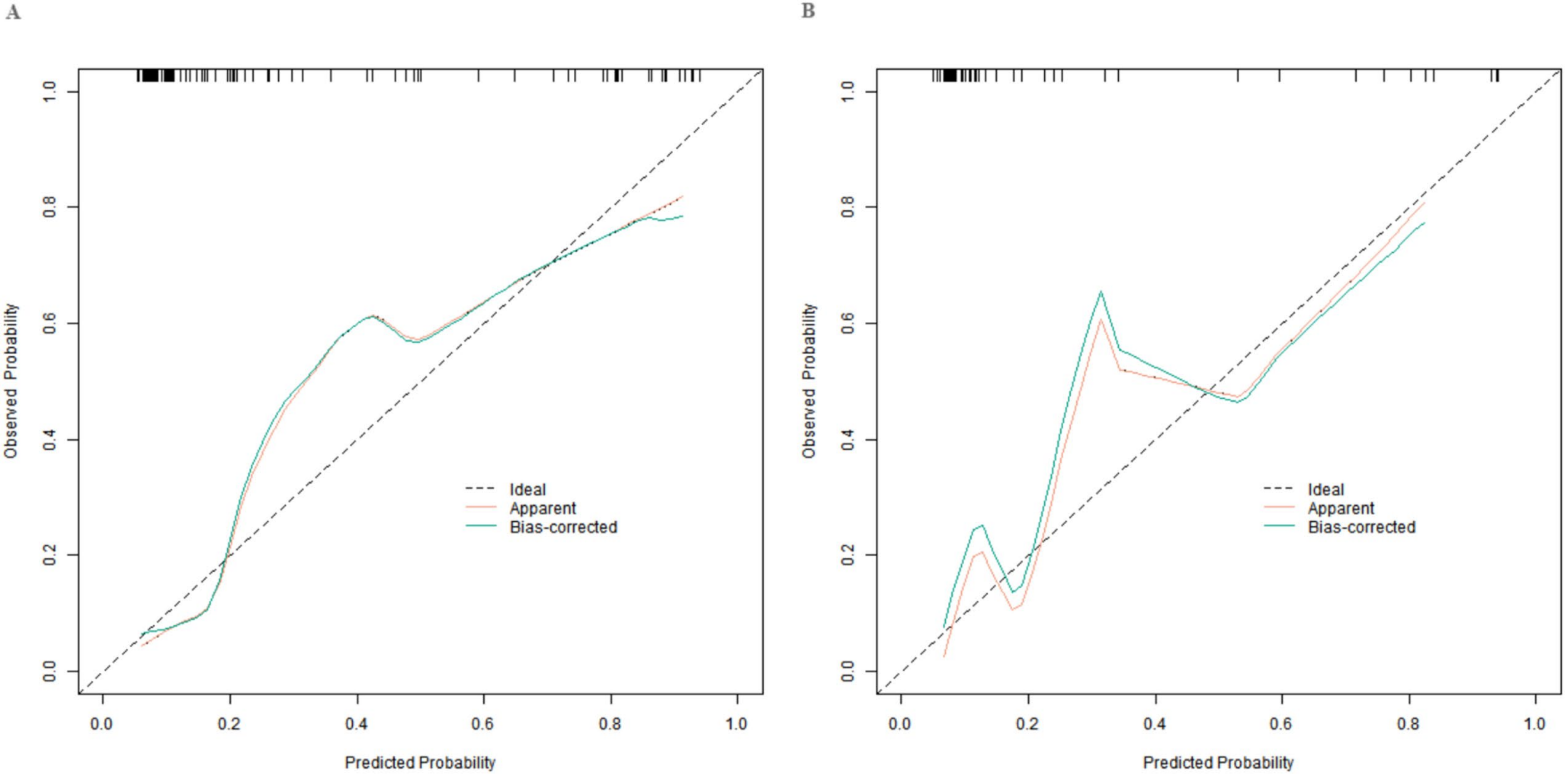
Calibration curves in the training set **(A)** and the validation set **(B)**.

**Figure 3:**
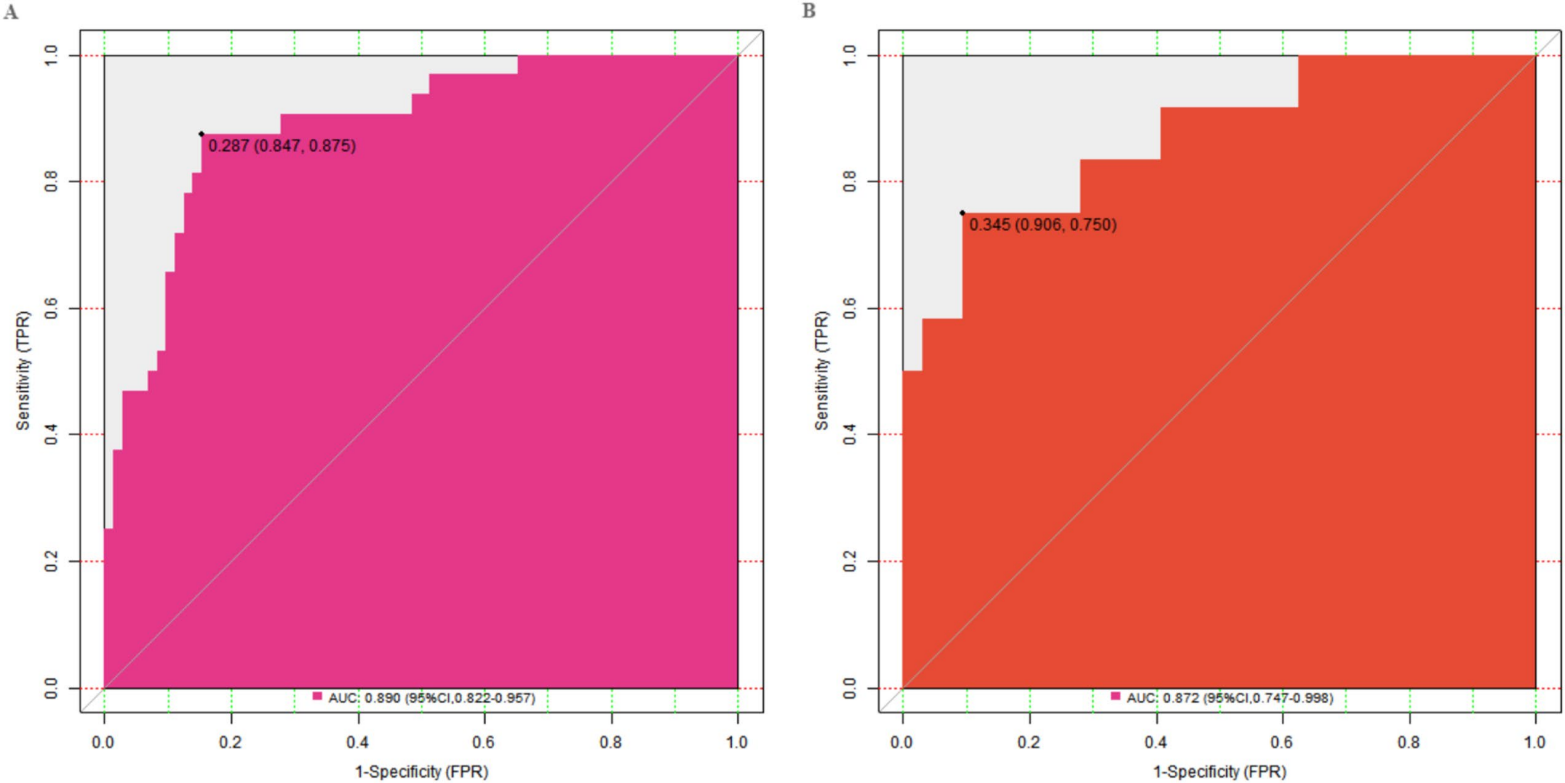
ROC curves in the training set **(A)** and the validation set **(B)**.

In this study, a standard model development and validation process was adopted. In the training set and the validation set, the C-index values of the constructed Nomogram prediction model were 0.890 and 0.872, respectively. Further analysis through calibration curves showed that there was a good consistency between the model-predicted values and the actual observed values, with the mean absolute errors being 0.124 and 0.114, respectively. Furthermore, the results of the Hosmer-Lemeshow test indicated that the χ^2^ values of the training set and the validation set were 9.907 (*p* = 0.271) and 7.194 (*p* = 0.515) respectively (Figure 2). This suggested that the model had a good goodness-of-fit and could fit the actual data well. In addition, the ROC curve analysis revealed the ability of the Nomogram model to predict the treatment effect. The AUC values of the training set and the validation set were 0.890 (95% CI: 0.822–0.957) and 0.872 (95% CI: 0.747–0.998) respectively (Figure 3). The closer the AUC value is to 1, the higher the prediction accuracy of the model, indicating that the model had a good prediction performance. Meanwhile, the combinations of sensitivity and specificity in the training set and the validation set were 0.847, 0.875 and 0.812, 0.750, respectively. Different combinations of sensitivity and specificity have different application values in different clinical scenarios, and doctors can select appropriate thresholds to apply the model according to the actual situation.

### Decision curve analysis of the nomogram prediction model

**Figure 4:**
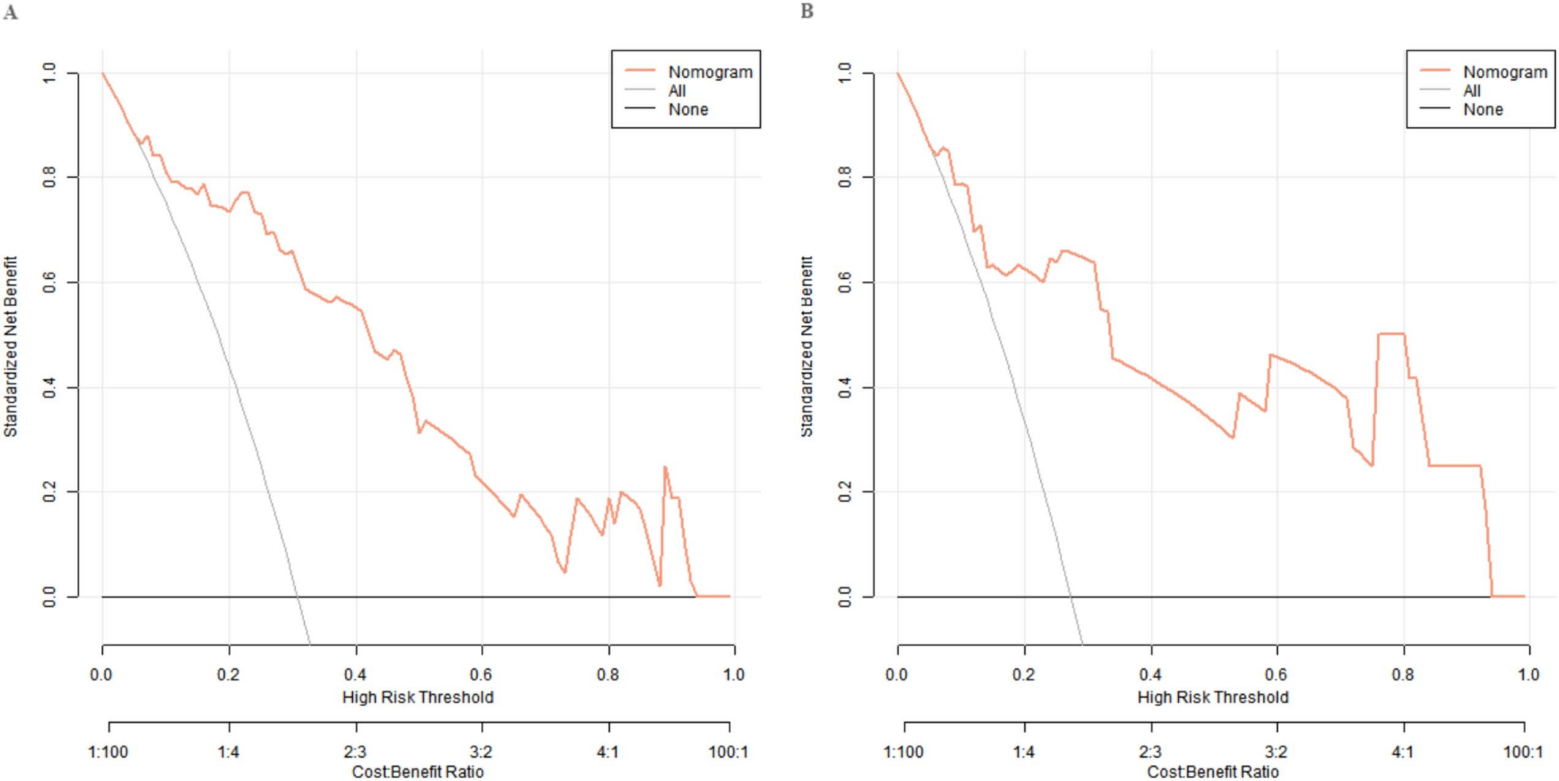
Decision curves in the training set **(A)** and the validation set **(B)**.

The decision curve showed that when the threshold probability is approximately between 0.06 and 0.95, the decision of applying the Nomogram model constructed in this study to predict the treatment effect has more clinical benefits compared with the decisions of considering the treatments as effective or all as ineffective. This indicates that the model can provide valuable references for clinical decision-making, assist doctors in more accurately evaluating patients’ treatment effects, and formulate more targeted personalized treatment plans (Figure 4).

## Discussion

Cerebral infarction, a common cerebrovascular disease, is characterized by high incidence, high disability rate, and high mortality (8). Lower limb dysfunctions one of its common sequelae, which seriously affects patients’ self-care ability and quality of life (9). Currently, conventional treatment regimens such as drug therapy and rehabilitation training, although serving as the basis for improving patients’ symptoms, have uneven effects (10). FES-MT training, as a new rehabilitation treatment method, promotes the recovery of patients’ lower limb motor function to a certain extent through electrical current stimulation of muscle contraction and mirror visual feedback (11). However, different patients respond differently to this combined treatment regimen, and the clinical effects are affected by multiple factors. In clinical practice, accurately predicting the treatment effect and formulating personalized treatment plans are crucial for improving patients’ prognosis. Currently, there is insufficient research on the influencing factors of the clinical effects of the combined treatment of conventional treatment regimens and FES-MT training for patients with lower limb dysfunction due to cerebral infarction, and there is a lack of accurate prediction models. This study aims to explore the influencing factors of the effect of the combined treatment of conventional treatment and FES-MT on lower limb dysfunction caused by cerebral infarction, construct and validate a Nomogram prediction model to assist in precise clinical treatment. A summary of the main findings of this study has showed (Tables 1–4).

### Influencing factors of treatment efficacy

In this study, multivariate Logistic regression analysis indicated that the time from onset to treatment, FPG, Hcy, infarct volume, NIHSS score, and pre-treatment Fugl - Meyer score (lower limb) were independent risk factors affecting the treatment effect.

The time from onset to treatment is one of the important factors affecting the treatment effect (12). As the time from onset to treatment prolongs, the difficulty of patients’ neurological function recovery increases, and the possibility of poor improvement in lower limb motor function also increases. Early treatment is crucial for saving damaged nerve cells and promoting neurological function recovery. In the acute phase after cerebral infarction, timely and effective rehabilitation treatment can better utilize the principle of neural plasticity to promote the remodeling and recovery of neurological function. Relevant studies have shown that patients who receive rehabilitation treatment within a few weeks after onset have significantly better motor function recovery than those with delayed treatment (13). In this study, the longer the time from onset to treatment, the higher the risk of ineffective treatment, which suggests that clinicians should shorten the time from onset to treatment as much as possible to improve the treatment effect. The FPG level is closely related to the treatment effect (14). Hyperglycemia has a toxic effect on nerve cells, affecting nerve conduction and repair, and thus hindering the recovery of lower limb motor function (15). Due to long-term hyperglycemia, diabetic patients have a higher incidence of neuropathy. When combined with lower limb dysfunction due to cerebral infarction, poor blood glucose control will further aggravate nerve damage. In this study, elevated FPG was an independent risk factor for poor treatment effect, which warns clinicians to closely monitor patients’ blood glucose levels during treatment, strictly control blood glucose, and strengthen blood glucose management for patients with diabetes to improve patients’ prognosis. As a sulfur-containing amino acid, elevated Hcy levels are closely related to the occurrence and development of cerebrovascular diseases (16). High levels of Hcy can cause vascular endothelial damage, enhance oxidative stress reactions, and increase the tendency of thrombosis through various mechanisms, thereby affecting the neurological function recovery of patients with cerebral infarction (17). In this study, elevated Hcy was an independent risk factor for ineffective treatment. The potential mechanisms by which Hcy impedes functional recovery are multifaceted. Primarily, high Hcy levels promote endothelial dysfunction and atherosclerosis, which can compromise cerebral perfusion and microcirculation, thereby hampering the delivery of oxygen and nutrients essential for neurorepair. Secondly, Hcy induces oxidative stress and excitotoxicity, leading to neuronal damage and apoptosis, which creates a hostile environment for neural plasticity. Finally, Hcy may interfere with neurotrophic factors and hinder the remodeling of neural circuits, which is the fundamental physiological process underpinning rehabilitation. Therefore, elevated Hcy creates a suboptimal biochemical and vascular environment that likely attenuates the brain’s capacity to respond to rehabilitative therapies like FES-MT. The infarct volume directly reflects the degree of brain tissue damage (18). A larger infarct volume means more nerve cell necrosis and loss of function, which will seriously affect the recovery of lower limb motor function (19). The more nerve conduction pathways involved in the infarct, the greater the impact on lower limb motor function (20). In this study, the larger the infarct volume, the worse the treatment effect, which provides an important basis for clinical evaluation of patients’ prognosis. For patients with a larger infarct volume, more aggressive rehabilitation treatment strategies and a longer rehabilitation period may be required. The NIHSS score is used to evaluate the degree of neurological deficit in patients, and a higher score indicates more severe neurological deficit (21). Severe neurological deficit will aggravate lower limb dysfunction and affect the effect of rehabilitation treatment (22). In this study, the NIHSS score was an independent risk factor for the treatment effect, which indicates that when formulating rehabilitation treatment plans, the degree of patients’ neurological deficit should be fully considered. For patients with a higher NIHSS score, targeted rehabilitation training should be strengthened to increase the possibility of lower limb motor function recovery. The pre-treatment lower limb Fugl-Meyer score is an important indicator for evaluating patients’ lower limb motor function, and a lower score indicates worse lower limb motor function (23, 24). Patients with poor pre-treatment basic motor function face greater challenges and have more difficulty in recovery during the rehabilitation treatment process. In this study, a low pre-treatment lower limb Fugl-Meyer score was associated with an increased risk of ineffective treatment, suggesting that clinicians should accurately evaluate patients’ lower limb motor function before treatment, formulate personalized rehabilitation treatment plans based on the score, and pay more attention and provide more interventions for patients with poor basic motor function. Notably, we also analyzed lesion location-a well-documented predictor of post-stroke motor recovery (25). Lesions in motor-related regions (e.g., internal capsule, primary motor cortex) can directly disrupt neural circuits underlying lower limb movement, theoretically reducing treatment responsiveness. However, our univariate analysis showed no significant difference in lesion location between the effective and ineffective groups (*p* = 0.628), which may be attributed to two factors: First, the relatively small sample size (*n* = 148 in the training set) may have limited statistical power to detect subtle differences in lesion location distribution. Second, the distribution of lesion locations in our cohort was relatively balanced, reducing the likelihood of group differences. Future studies with larger sample sizes could further explore whether combining lesion location with infarct volume improves the model’s predictive accuracy. Notably, this study included patients with a post-stroke interval of 1–6 months, which may introduce clinical heterogeneity. Motor recovery after stroke is most active in the first 3 months (subacute phase) due to strong neural plasticity, whereas recovery slows in the 4–6-month window (early chronic phase). This temporal difference may affect treatment responsiveness: patients in the 1–3-month group may have a lower risk of ineffective treatment than those in the 4–6-month group. Although the multivariate analysis adjusted for this factor, future studies could stratify patients by post-stroke interval (e.g., <3 months vs. 3–6 months) to optimize the model’s specificity for different recovery stages.

### Performance of the nomogram model

The Nomogram prediction model constructed based on the above independent risk factors had C-index values of 0.890 and 0.872 in the training set and validation set respectively, and mean absolute errors of 0.124 and 0.114, respectively. The closer the C-index is to 1, the better the model’s discrimination ability, indicating that the model has a good ability to predict the treatment effect. The mean absolute error reflects the average deviation between the model’s predicted values and the actual values. A lower mean absolute error indicates good consistency between the model’s predicted values and the actual observed values. In the Hosmer-Lemeshow test, the χ^2^ values of the training set and validation set were 9.907 (*p* = 0.271) and 7.194 (*p* = 0.515) respectively. A *p*-value greater than 0.05 indicates a good goodness-of-fit of the model, which can fit the actual data well, that is, the model can better reflect the relationship between various factors and the treatment effect. The ROC curve showed that the AUC values of the training set and validation set were 0.890 (95% CI: 0.822–0.957) and 0.872 (95% CI: 0.747–0.998) respectively. The closer the AUC value is to 1, the higher the prediction accuracy of the model, further proving that the model has good prediction performance. The combinations of sensitivity and specificity in the training set and validation set were 0.847, 0.875 and 0.812, 0.750, respectively. Different combinations of sensitivity and specificity have different application values in different clinical scenarios. When it is necessary to avoid missed diagnoses as much as possible, a threshold with higher sensitivity can be selected; when it is necessary to reduce misdiagnoses, a threshold with higher specificity can be selected. Doctors can select appropriate thresholds according to the actual situation to apply the model, providing valuable references for clinical decision-making.

### Limitations and future directions

The Nomogram prediction model constructed in this study integrates multiple influencing factors and can intuitively predict the probability of ineffective treatment for patients, providing a new tool for clinicians to evaluate the treatment effect and formulate personalized treatment plans. Through this model, doctors can comprehensively consider various factors of patients before treatment, predict the treatment effect, adjust the treatment plan for high-risk patients, increase the treatment intensity, or adopt more targeted treatment methods, thereby improving the precision and effectiveness of treatment and improving patients’ prognosis. However, this study also has certain limitations. Firstly, the sample size is relatively limited, which may affect the stability and universality of the model. Subsequent studies can expand the sample size to further verify the accuracy and reliability of the model. Secondly, this study is a single-center study, and the patient source is relatively single, lacking data from patients in different regions and different ethnic groups, which may lead to certain biases in the research results. Future studies can conduct multi-center, large-sample research to improve the representativeness of the research results. In addition, this study did not conduct long-term follow-up on patients, so it is impossible to evaluate the long-term stability of the treatment effect and the changes of influencing factors in the long-term process. In future studies, long-term follow-up should be added to more comprehensively evaluate the treatment effect and influencing factors. Finally, this study was not an RCT without a control group, with all patients receiving conventional therapy combined with FES-MT. This prevents isolating FES-MT’s independent efficacy from the overall regimen. Moreover, though conventional treatments were standardized, detailed information on other concurrent therapies was not collected, potentially introducing confounding factors. Future RCTs with control groups are needed to clarify FES-MT’s independent role, with detailed recording of all concurrent treatments.

It is important to consider the context of the presented interventions. All patients in this study received a comprehensive and standardized conventional rehabilitation program alongside FES-MT. Therefore, the predictive model developed herein does not isolate the effect of FES-MT alone but rather identifies factors associated with a favorable response to a combined treatment regimen that includes FES-MT. The influencing factors we identified (e.g., time from onset, infarct volume, baseline function) likely reflect a patient’s inherent capacity for recovery within this specific multimodal therapeutic framework. Consequently, the nomogram is clinically valuable for predicting which patients are most likely to benefit from this integrated approach, aiding in personalized treatment planning and resource allocation.

This study screened out independent influencing factors affecting the treatment effect through research on patients with lower limb dysfunction due to cerebral infarction treated with the combined treatment of conventional treatment and FES-MT, and constructed a Nomogram prediction model with good prediction performance. This model provides a valuable reference tool for clinicians, but it still needs further improvement and validation. It is hoped that there will be more high-quality studies in the future to provide more precise and effective treatment plans for patients with lower limb dysfunction due to cerebral infarction and improve their quality of life.

## Data Availability

The data supporting this study are available from the corresponding author upon reasonable request.

## References

[1] Nguyen NB Nguyen Thi HH Thi HL Nguyen ST Nguyen TV. Results of acute cerebral infarction treatment with hyperbaric oxygen therapy, 2020-2022. Int Marit Health. (2023) 74:265–71. doi: 10.5603/imh.97720, PMID: 38111247

[2] Purrahman D Shojaeian A Poniatowski ŁA Piechowski-Jóźwiak B Mahmoudian-Sani MR. The role of Progranulin (PGRN) in the pathogenesis of ischemic stroke. Cell Mol Neurobiol. (2023) 43:3435–47. doi: 10.1007/s10571-023-01396-8, PMID: 37561339 PMC11410000

[3] Jia DM Li X Zhang BC Zhang BR Zhang QJ Liu MW . Therapeutic efficacy of repetitive transcranial magnetic stimulation on gait and limb balance function in patients with lower limb dysfunction post-cerebral infarction: a systematic review and meta-analysis. BMC Neurol. (2025) 25:126. doi: 10.1186/s12883-025-04112-9, PMID: 40128695 PMC11931868

[4] Geng H Zhang L Xin C Zhang C Xie Y. Xuesaitong oral preparation as adjuvant therapy for treating acute cerebral infarction: a systematic review and meta-analysis of randomized controlled trials. J Ethnopharmacol. (2022) 285:114849. doi: 10.1016/j.jep.2021.114849, PMID: 34800648

[5] Kim YS Song JY Park SH Lee MM. Effect of functional electrical stimulation-based mirror therapy using gesture recognition biofeedback on upper extremity function in patients with chronic stroke: a randomized controlled trial. Medicine (Baltimore). (2023) 102:e36546. doi: 10.1097/MD.0000000000036546, PMID: 38206692 PMC10754587

[6] Hernández ED Forero SM Galeano CP Barbosa NE Sunnerhagen KS Alt Murphy M. Intra- and inter-rater reliability of Fugl-Meyer assessment of lower extremity early after stroke. Braz J Phys Ther. (2021) 25:709–18. doi: 10.1016/j.bjpt.2020.12.002, PMID: 33358073 PMC8721065

[7] Alemseged F Rocco A Arba F Schwabova JP Wu T Cavicchia L . Posterior national institutes of health stroke scale improves prognostic accuracy in posterior circulation stroke. Stroke. (2022) 53:1247–55. doi: 10.1161/STROKEAHA.120.034019, PMID: 34905944

[8] Ng TP Wong C Leong ELE Tan BY Chan MYY Yeo LL . Simultaneous cardio-cerebral infarction: a meta-analysis. QJM. (2022) 115:374–80. doi: 10.1093/qjmed/hcab158, PMID: 34051098

[9] Zhu ZL Shen TY Sun Z Li H Shan H Cao LL . Effects of zhongfeng cutong moxibustion on motor function and corticospinal tract in the patients with motor dysfunction during the recovery period of cerebral infarction. Zhongguo Zhen Jiu. (2023) 43:1358–62. doi: 10.13703/j.0255-2930.20230623-k0003, PMID: 38092532

[10] Xia J Pei S Chen Z Wang L Hu J Wang J. Effects of conventional speech therapy with Liuzijue qigong, a traditional Chinese method of breath training, in 70 patients with post-stroke spastic dysarthria. Med Sci Monit. (2023) 29:e939623. doi: 10.12659/MSM.939623, PMID: 37365796 PMC10314716

[11] Li P Xin H Zhang X Li X Dou L Wang G . Effect of MVF combined with FES on limb function recovery and fine function rehabilitation of hemiplegic patients after ACI. Altern Ther Health Med. (2024) 30:212–9. 38430148

[12] Jovin TG Nogueira RG Lansberg MG Demchuk AM Martins SO Mocco J . Thrombectomy for anterior circulation stroke beyond 6 h from time last known well (AURORA): a systematic review and individual patient data meta-analysis. Lancet. (2022) 399:249–58. doi: 10.1016/S0140-6736(21)01341-6, PMID: 34774198

[13] Nogueira RG Doheim MF Jadhav AP Aghaebrahim A Frankel MR Jankowitz BT . Mode of onset modifies the effect of time to endovascular reperfusion on clinical outcomes after acute ischemic stroke: an analysis of the DAWN trial. Ann Neurol. (2024) 96:356–64. doi: 10.1002/ana.26968, PMID: 38877793

[14] Jia J Wang L Zhang L Hong Z Xia R Zhao Z . Analysis of the expression levels of chemerin, ox-LDL, MMP-9, and PAPP-A in ICVD patients and their relationship with the severity of neurological impairment. Brain Behav. (2022) 12:e2613. doi: 10.1002/brb3.2613, PMID: 35620813 PMC9304843

[15] Yang Y Zhao B Wang Y Lan H Liu X Hu Y . Diabetic neuropathy: cutting-edge research and future directions. Signal Transduct Target Ther. (2025) 10:132. doi: 10.1038/s41392-025-02175-1, PMID: 40274830 PMC12022100

[16] Wang L Wang ZH Liu LP. Value of Hcy combined with Framingham score for predicting macrovascular disease in elderly patients with type 2 diabetes. Medicine (Baltimore). (2023) 102:e35401. doi: 10.1097/MD.0000000000035401, PMID: 37800767 PMC10553110

[17] Li Z Wu X Huang H Xu F Liang G Lin C . MTHFR C677T polymorphism and cerebrovascular lesions in elderly patients with CSVD: a correlation analysis. Front Genet. (2022) 13:987519. doi: 10.3389/fgene.2022.987519, PMID: 36212120 PMC9537945

[18] Wu C Wu C Peng L Wu M Li Z Chen J. Multi-omics approaches for the understanding of therapeutic mechanism for Huang-qi-long-Dan granule against ischemic stroke. Pharmacol Res. (2024) 205:107229. doi: 10.1016/j.phrs.2024.107229, PMID: 38782148

[19] Mo J Liao W Du J . Buyang huanwu decoction improves synaptic plasticity of ischemic stroke by regulating the cAMP/PKA/CREB pathway. J Ethnopharmacol. (2024) 335:118636. doi: 10.1016/j.jep.2024.118636, PMID: 39089658

[20] Liu Y Li Y Zang J Zhang T Li Y Tan Z . CircOGDH is a penumbra biomarker and therapeutic target in acute ischemic stroke. Circ Res. (2022) 130:907–24. doi: 10.1161/CIRCRESAHA.121.319412, PMID: 35189704

[21] Rahnemayan S Ala A Taghizadeh N Sadeghi-Hokmabadi E Entezari I Shams Vahdati S. Shortened NIHSS for rapid stroke assessment in emergency care settings. Neurologist. (2024) 30:150–4. doi: 10.1097/NRL.0000000000000608, PMID: 39722575

[22] Lima JPS Silva LA Delisle-Rodriguez D Cardoso VF Nakamura-Palacios EM Bastos-Filho TF. Unraveling transformative effects after tDCS and BCI intervention in chronic post-stroke patient rehabilitation-an alternative treatment design study. Sensors (Basel). (2023) 23:9302. doi: 10.3390/s23239302, PMID: 38067674 PMC10708803

[23] Wang F Zhang S Zhou F Zhao M Zhao H. Early physical rehabilitation therapy between 24 and 48 h following acute ischemic stroke onset: a randomized controlled trial. Disabil Rehabil. (2022) 44:3967–72. doi: 10.1080/09638288.2021.1897168, PMID: 33736542

[24] Poniatowski ŁA Cudna A Kurczych K Bronisz E Kurkowska-Jastrzębska I. Kinetics of serum brain-derived neurotrophic factor (BDNF) concentration levels in epileptic patients after generalized tonic-clonic seizures. Epilepsy Res. (2021) 173:106612. doi: 10.1016/j.eplepsyres.2021.106612, PMID: 33774427

[25] Bowren M Bruss J Manzel K Edwards D Liu C Corbetta M . Post-stroke outcomes predicted from multivariate lesion-behaviour and lesion network mapping. Brain. (2022) 145:1338–53. doi: 10.1093/brain/awac010, PMID: 35025994 PMC9630711

